# A spatiotemporal comparison of length-at-age in the coral reef fish *Acanthurus nigrofuscus* between marine reserves and fished reefs

**DOI:** 10.1371/journal.pone.0239842

**Published:** 2020-09-28

**Authors:** Mathias T. Cramer, Robert Y. Fidler, Louis M. Penrod, Jessica Carroll, Ralph G. Turingan

**Affiliations:** 1 Department of Ocean Engineering and Marine Sciences, Florida Institute of Technology, Melbourne, Florida, United States of America; 2 Department of Biological Sciences, Florida International University, Miami, Florida, United States of America; 3 Fish and Wildlife Research Institute, Florida Fish and Wildlife Conservation Commission, St. Petersburg, Florida, United States of America; Swansea University, UNITED KINGDOM

## Abstract

Quantitative assessments of the capacity of marine reserves to restore historical fish body-size distributions require extensive repeated sampling to map the phenotypic responses of target populations to protection. However, the “no take” status of marine reserves oftentimes precludes repeated sampling within their borders and, as a result, our current understanding of the capacity of marine reserves to restore historical body-size distributions remains almost entirely reliant on independent, static visual surveys. To overcome this challenge, we promote the application of a traditional fisheries tool known as a “back-calculation”, which allows for the estimation of fish body lengths from otolith annuli distances. This practical application was pursued in this study, using data collected in five marine reserves and adjacent fished reefs in the Philippines, to investigate spatiotemporal disparities in length-at-age of the brown surgeonfish, *Acanthurus nigrofuscus*. The spatial component of our analyses revealed that 1) *A*. *nigrofuscus* were phenotypically similar between marine reserves and fished reefs during their early life history; 2) marine reserve and fished reef populations diverged into significantly different length-at-age morphs between ages three and six, in which protected fish were predominantly larger than conspecifics in fished reefs; and 3) *A*. *nigrofuscus* returned to a state of general phenotypic similarity during later life. The temporal component of our analyses revealed that younger generations of *A*. *nigrofuscus* exhibited significant, positive year effects that were maintained until age eight, indicating that, within the significant age cohorts, younger generations were significantly larger than older generations.

## Introduction

Reductions in length-at-age are well known to diminish population fitness and the sustainability of fisheries stocks through consequential effects on reproduction and survival [1–5]. In most fishes, larger body sizes confer greater survival probability, reproductive output, and offspring viability [6, 7], while smaller body sizes are highly correlated with the opposite [8–12]. Marine reserves (MRs), which are geographical marine areas that have been designated by a governing authority to strengthen the long-term sustainability of the contained natural resources through the prohibition of exploitative activities [13], should allow for the restoration of historical body-size distributions by (1) eliminating size-selective fishing mortality and (2) resuming ecological drivers that naturally promote larger body sizes [14–17]. In turn, it is expected that protected populations will subsidize adjacent fished reefs (FRs) through larval seeding and post-settlement spillover [15, 18–20]. While many studies have found beneficial biophysical developments within MRs compared to FRs, including but not limited to increased biomass, biodiversity, body-size distributions and coral recruitment [18, 21–26], definitive assessments of MR functionality require extensive repeated sampling. Unfortunately, the “no take” status of MRs oftentimes precludes repeated sampling within their borders and, as a result, our current understanding of the capacity of MRs to restore historical body-size distributions remains almost entirely reliant on independent, static visual surveys. To overcome this challenge, we promote the application of a traditional fisheries tool known as a “back-calculation”.

A back-calculation allows for the estimation of fish body lengths from sagittal otolith (ear stone) annuli distances, thereby providing a method by which length-at-age data from earlier time periods in the life history of a fish can be obtained from a single sampling effort [27]. Traditionally, the primary function of back-calculations has been to ease the attainment of length-at-age data in order to construct growth curves, which are used extensively in the fields of aquaculture and fisheries management. Additionally, as illustrated for the first time in this paper, these approaches can be readily adopted into analyses of MR functionality as growth models elucidate shifting demographics and phenotypes, providing insight into a population’s specific density-dependent scenario [28]. This information not only allows for a more accurate examination of MR performance, but it also enhances our ability to inform fisheries managers of potential adaptive management interventions.

In this study, we performed static samplings between five MRs and adjacent FRs in the Philippines to introduce the application of a back-calculation model into the field of MR assessment. The brown surgeonfish, *Acanthurus nigrofuscus* (Acanthuridae, Teleostei), was used as a representative species of the Acanthuridae family that has critical anthropogenic and ecological importance in the Coral Triangle region. Investigations estimate that near-shore fisheries, of which acanthurids comprise a major component, sustain the primary source of dietary protein for approximately one billion people in Asia [29]. In addition, acanthurids perform the critical ecological role of mitigating macroalgal colonization on coral reefs [30–32]. As a result, reductions in the functional group’s regional biomass could interfere with their capacity to adequately mitigate algal growth and consequently diminish coral reef resilience [33, 34]. Nonetheless, there remains a major gap in our current knowledge of acanthurid species’ populational health in Southeast Asia. These concerns are intensified in the wake of analyses that determined, via the application of the International Union for Conservation of Nature–Species Survival Commission’s Susceptibility Matrix, that many acanthurid species exhibit extrinsic as well as intrinsic traits that make them particularly susceptible to localized extirpation [35].

Here, length-at-age data obtained through otolith-based back-calculations were used to construct age-specific growth models that allowed for in-depth spatiotemporal comparisons of *A*. *nigrofuscus*’ length-at-age performance between MRs and FRs in the Philippines. Assuming that the beneficial biophysical developments previously discussed and characteristically noted by past MR assessments are applicable here, we hypothesize that MR populations will exhibit greater lengths-at-age than FR populations and that newer generations will exhibit greater lengths-at-age than older generations.

## Materials and methods

### Sampling locations and permits

**Fig 1:**
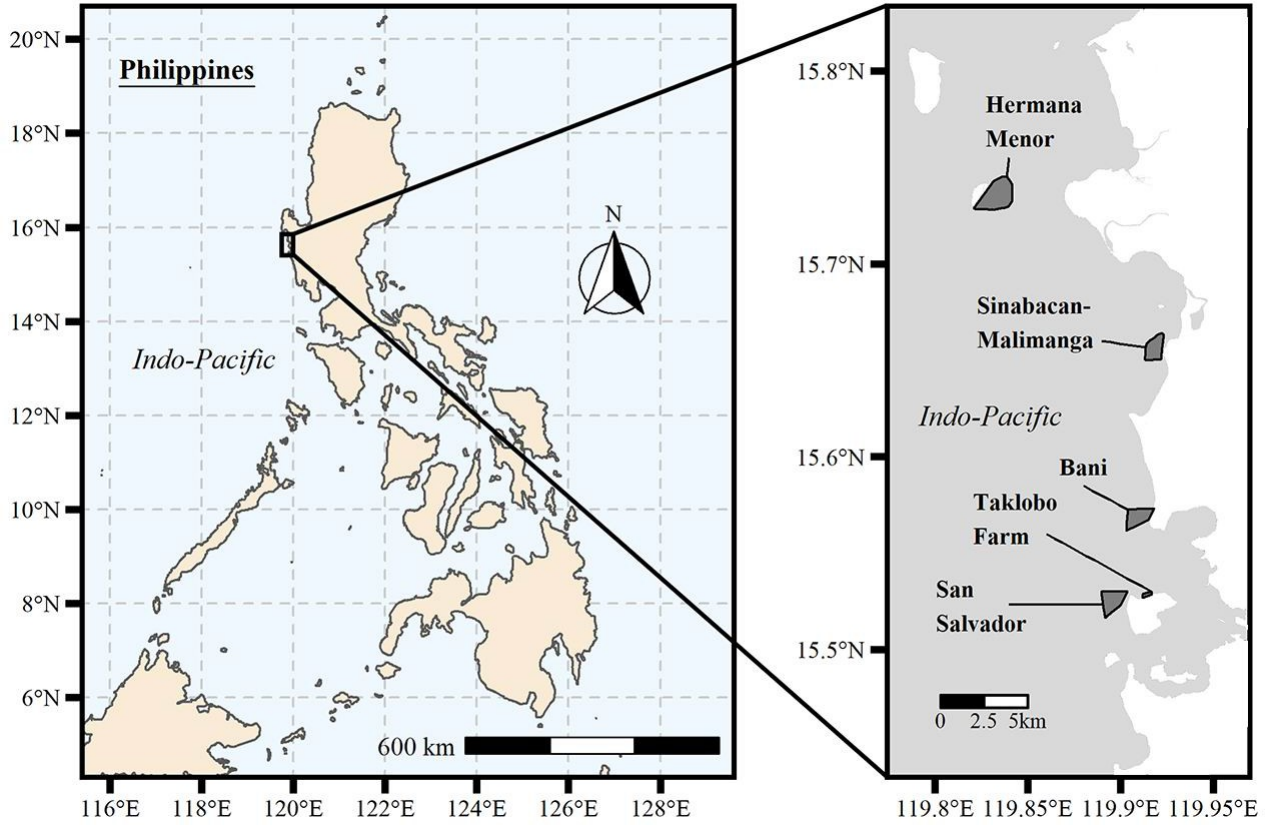
A) A map of the Philippines and B) the five MR locations studied in the Zambales Province of Luzon. The map data used for Fig 1A was obtained from Natural Earth through the *rnaturalearth* [45] and *rnatrualearthhires* [46] packages in R. The map tiles used for Fig 1B are from Stamen Design, under CC BY 3.0 with data by OpenStreetMap, under ODbL and obtained through the *ggmap* [41] package.

Specimens were collected in 2015 across five strongly enforced MRs and adjacent FRs that spanned three municipalities within the Zambales Province of Luzon, Philippines: 1. Sinabacan-Malimanga MR (Municipality of Candelaria [est. 2011; 124 ha]); 2. Hermana Menor MR (Municipality of Santa Cruz [est. 2003; 266 ha]); 3. Bani MR (Municipality of Masinloc [est. 2006; 50 ha]); 4. San Salvador MR (Municipality of Masinloc [est. 1989; 127 ha]); and 5. Taklobo Farm (Municipality of Masinloc [est. 1989; 2 ha]) (Fig 1). All FR specimens were collected at least 300 meters away from MR boundaries. Collection was targeted with the intention of sampling across the observed body-size range. Fig 1 was constructed using R v. 3.5.0. [36] using the *mapview* [37], *sp* [38], *sf* [39], *ggplot2* [40], *ggmap* [41], *ggsn* [42], *ggspatial* [43], *patchwork* [44], *rnaturalearth* [45] and *rnaturalearthhires* [46] packages.

Approvals from the Department of Agriculture of the Philippines, the Bureau of Fisheries and Aquatic Resources of the Philippines and the local governmental units of Candelaria, Masinloc and Santa Cruz were obtained. All vertebrate work was independently reviewed and approved by the Institutional Animal Care and Use Committees of the Florida Institute of Technology and the University of the Philippines. Sampling procedures were additionally reviewed and approved as part of obtaining the Philippines, Department of Agriculture Gratuitous Permit GP-0096-15 issued on June 01, 2015.

### Length and otolith dissections

Sampling was performed via scuba with spearfishing equipment. Following collection, specimens were euthanized via ice immersion and transported back to a laboratory where standard length (mm) measurements were taken. Sagittal otoliths were removed and prepared for ageing using the protocol described in Fidler et al. [17]. Ageing was performed in collaboration with the Fish and Wildlife Research Institute of the Florida Fish and Wildlife Conservation Commission.

### Modified fry back-calculation model

Various independent validation assessments have determined that the modified fry back-calculation (MFBC) model used in this study operates as an efficient and conservative model for the back-calculation of body lengths from annuli distances [47, 48]. In addition, longitudinal in-aquaria growth experiments determined that the MFBC model exhibited an incredible robustness in the procurement of accurate, precise and unbiased length-at-age data, despite exposing specimens to differing extrinsic factors known to affect somatic growth [49]. Lastly, critiques that the proportional relationship between the growth of an individual’s body and otolith may fluctuate throughout the life history and consequently render back-calculations ineffective have been debunked following validation from Vigliola et al. [47], who confirmed the MFBC model’s capacity to accommodate such shifts in proportionality. The back-calculated length (*L*) of a fish at age (*i*) was calculated as:

$$L_{i}=a+\exp\;(\ln\;(L_{0P}-a)+\frac{\left[\ln\;\left(L_{cpt}–a\right)–\ln\;(L_{0P}–a)][ln\;(R_{i})–ln(R_{0P})\right]}{\left[\ln\;(R_{cpt})–\ln\left(R_{0P}\right)\right)})$$

where *L*_*cpt*_ is the length of a fish at capture, *R*_*cpt*_ is the radius of a fish’s otolith at capture, *a* is the biological intercept between *L*_*cpt*_ and *R*_*cpt*_, *L*_*0P*_ is the length of a fish at increment formation, *R*_*i*_ is the otolith radius at age *i*, and *R*_*0P*_ is the mean radius of the first annulus in the otolith.

**Fig 2:**
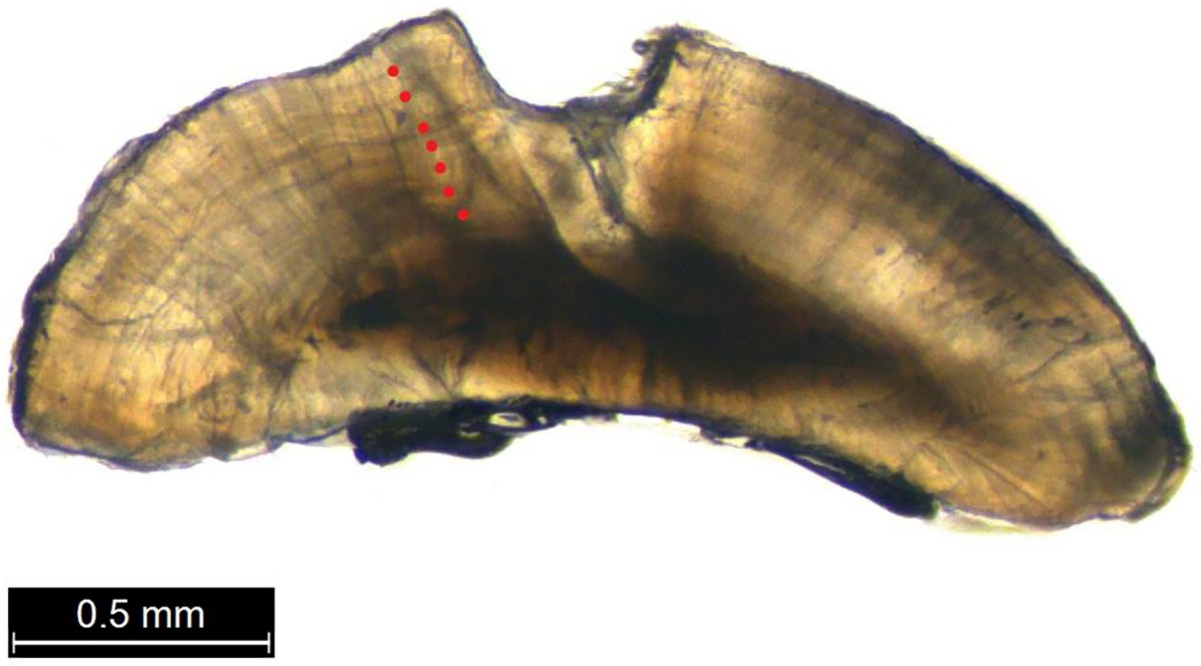
The left sagittal otolith of a seven-year-old *A*. *nigrofuscus*. Red dots represent the seven annuli rings.

ImageJ1 [50] was used to obtain incremental radial measurements, defined as the distance from the nucleus of an otolith to the end of any given annulus, from photographs of specimens’ left sagittal otoliths. The measurement pane along which incremental radial measurements were taken was standardized at an approximate 45 degrees (Fig 2). The MFBC model was coded and applied using R v. 3.5.0. [36]. Packages required were: *FSA* [51] and *openxlsx* [52]. The total number of specimens collected within each age cohort are presented alongside the updated, back-calculated sample sizes in Table 1.

**Table 1:** Comparison of sample sizes between the original stocks of fish used for otolith ageing and the pooled stocks of fish that resulted from the MFBC model.

Data	Status	Age 1	Age 2	Age 3	Age 4	Age 5	Age 6	Age 7	Age 8	Age 9	Age 10	Age 11	Age 12
Original Sample Size	MR	30	24	11	10	9	9	5	3	4	3	1	2
	FR	31	25	28	21	20	18	5	1	1	6	2	1
Back-Calculated Sample Size	MR	111	81	57	46	36	27	18	13	10	6	3	2
	FR	159	128	103	75	54	34	16	11	10	9	3	1

### Statistical analyses

Similarities and disparities in length-at-age were statistically analyzed via two-way analyses of variance (ANOVAs), which allowed for examination of (1) age-specific differences between MR and FR populations (referred to as “status effects”), as well as (2) age-specific differences between year classes (referred to as “year effects”). A posteriori power analysis was additionally performed to determine the statistical power of our spatial analyses (status effects). Results are presented as locally estimated scatterplot smoothing regressions (LOESS: a robust non-parametric regression method) with 95% confidence intervals. LOESS span values, which determine the degree of regression smoothing, were standardized at 0.95 to focus and emphasize visualization of the large-scale phenotypic shifts examined in this study. All statistics and figures were computed using R v. 3.5.0. [36]. Packages required were: *pwr* [53], *car* [54], *userfriendlyscience* [55] and *ggplot2* [40].

## Results

**Fig 3:**
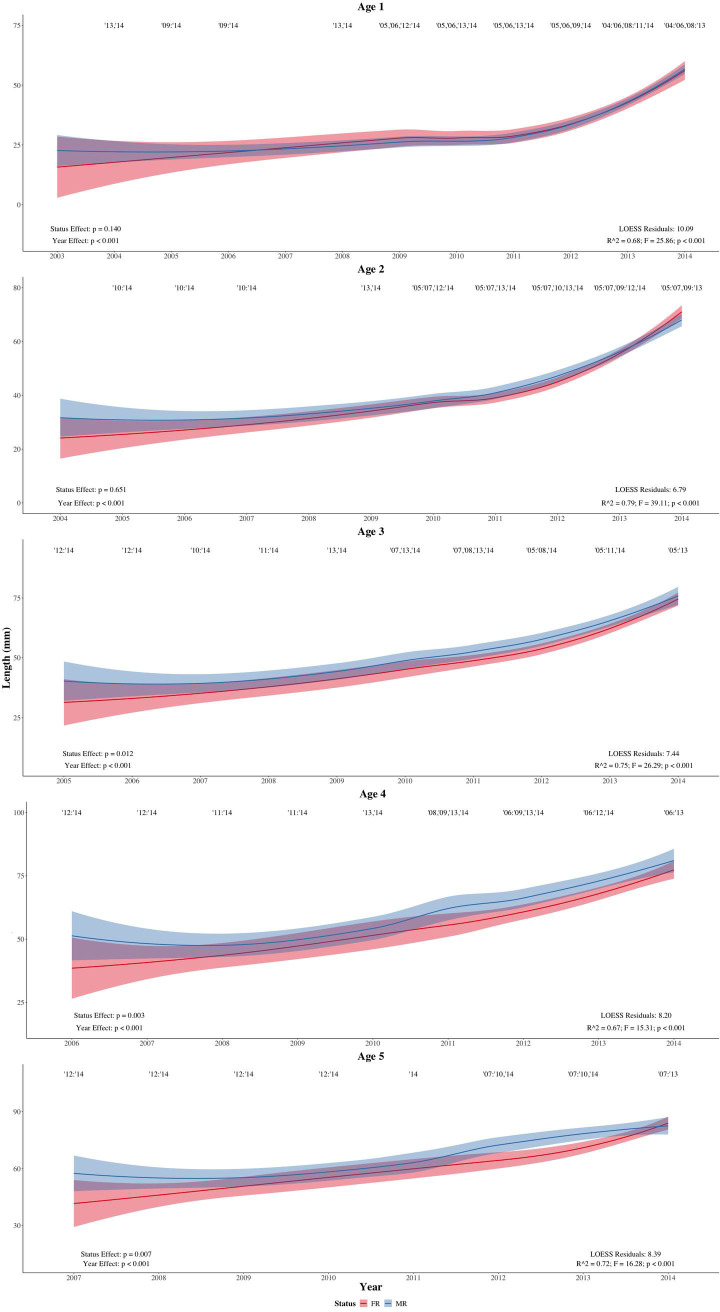
LOESS regressions for age cohorts one to five. Two-way ANOVA results are shown in the lower left corner of each age plot, with significant disparities between year classes (year effects) indicated via year numbers above the regressions of each age plot.

**Fig 4:**
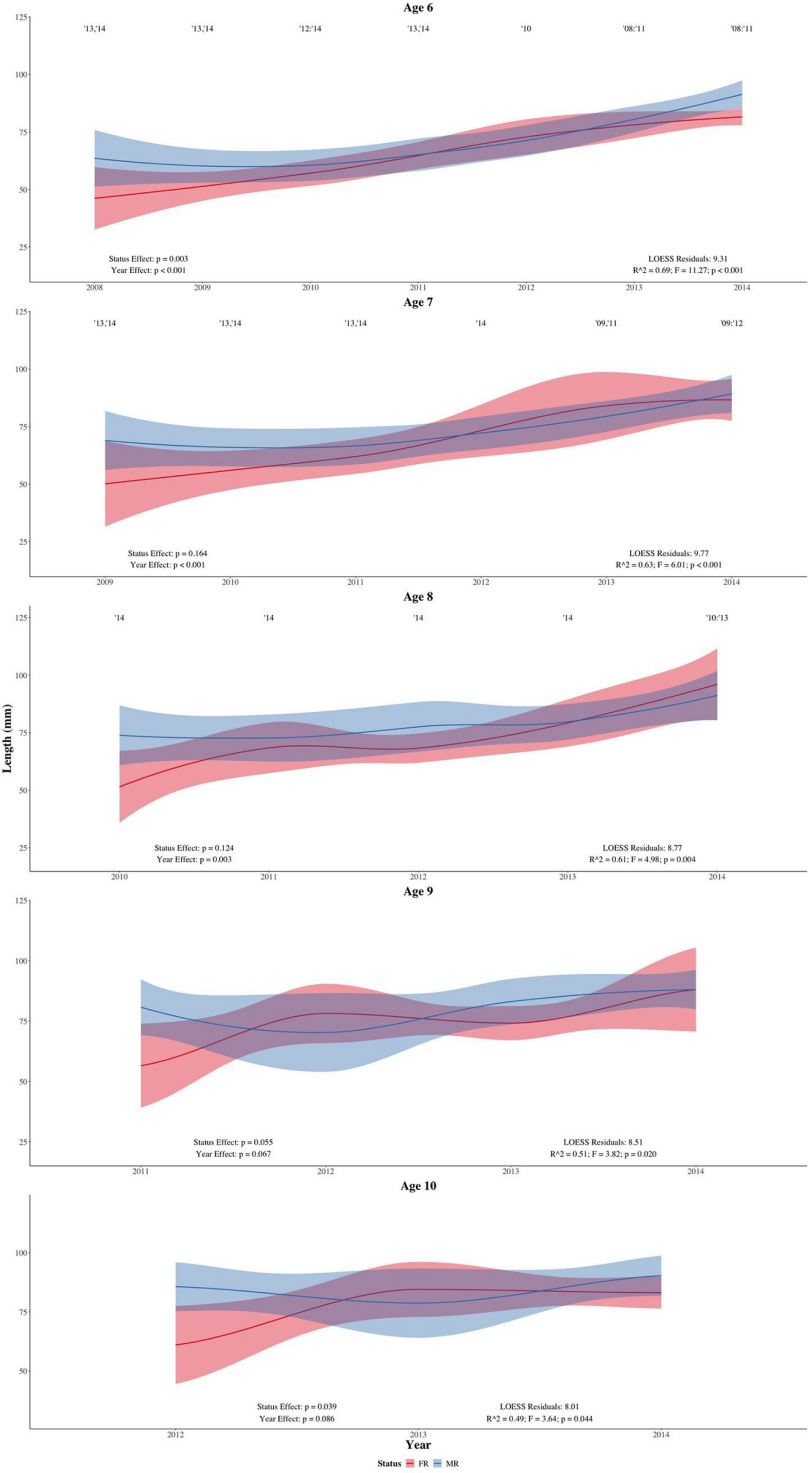
LOESS regressions for age cohorts six to ten. Two-way ANOVA results are shown in the lower left corner of each age plot, with significant disparities between year classes (year effects) indicated via year numbers above the regressions of each age plot.

Significant disparities in the body lengths of MR and FR populations were found for five age cohorts (two-way ANOVA). No significant status effect was found at ages one and two (F = 2.19, p = 0.140; F = 0.21, p = 0.651, respectively) although an effect appeared at ages three, four, five and six (F = 6.55, p = 0.012; F = 9.16, p = 0.003; F = 7.65, p = 0.007; F = 9.65, p = 0.003, respectively; Figs 3 and 4). Significant disparity disappeared at ages seven, eight and nine (F = 2.08, p = 0.164; F = 2.68, p = 0.124; F = 4.50, p = 0.055, respectively) but reappeared at age ten (F = 5.81, p = 0.039; Fig 4). No significant status effect was detected at age 11 (F = 2.48, p = 0.256), as shown in Table 2. A posteriori power analysis identified low statistical power across our spatial analyses (Table 2).

**Table 2:** *A*. *nigrofuscus* mean lengths and standard errors of the mean in millimeters for each age cohort separated by management status, along with two-way ANOVA F-values (F), degrees of freedom (DF), and p-values (p) for both status and year effects. Power of each age cohorts’ spatial analysis is additionally presented. Significant values indicated in bold.

	Age 1	Age 2	Age 3	Age 4	Age 5	Age 6	Age 7	Age 8	Age 9	Age 10	Age 11	Age 12
**MR**												
Mean	38.55	49.57	56.90	65.36	71.46	76.58	76.53	79.47	83.27	86.84	87.60	93.00
Standard Error	1.36	1.71	1.87	2.06	2.11	2.90	2.86	2.84	2.41	2.37	1.31	4.00
**FR**												
Mean	36.14	48.46	57.89	64.50	70.96	73.10	70.34	70.41	74.54	81.01	83.37	78.00
Standard Error	1.33	1.36	1.45	1.60	2.00	2.40	3.96	3.56	3.01	3.18	8.73	NA
**Difference**												
Absolute	2.41	1.11	0.99	0.86	0.50	3.48	6.19	9.06	8.73	5.83	4.23	15.00
Percent	6.46	2.27	1.73	1.33	0.70	4.65	8.43	12.08	11.07	6.95	4.95	17.54
**Status**												
F	2.19	0.21	6.55	9.16	7.65	9.65	2.08	2.68	4.50	5.81	2.48	NA
DF	1	1	1	1	1	1	1	1	1	1	1	NA
p	0.140	0.651	**0.012**	**0.003**	**0.007**	**0.003**	0.164	0.124	0.055	**0.039**	0.256	NA
Power	0.23	0.08	0.07	0.06	0.05	0.15	0.23	0.44	0.49	0.23	0.07	NA
**Year**												
F	53.32	81.04	54.28	31.09	32.23	22.16	10.64	6.91	3.11	3.27	4.82	NA
DF	11	10	9	8	7	6	5	4	3	2	1	NA
p	**< 0.001**	**< 0.001**	**< 0.001**	**< 0.001**	**< 0.001**	**< 0.001**	**< 0.001**	**0.003**	0.067	0.086	0.159	NA

Significant year effects were abundant throughout the observed life history, appearing between ages one and eight (F = 53.32, p < 0.001; F = 81.04, p < 0.001; F = 54.28, p < 0.001; F = 31.09, p < 0.001; F = 32.23, p < 0.001; F = 22.16, p < 0.001; F = 10.64, p < 0.001; F = 6.91, p = 0.003, respectively; Figs 3 and 4). No significant year effect was detected at ages nine, ten and 11 (F = 3.11, p = 0.067; F = 3.27, p = 0.086; F = 4.82, p = 0.159, respectively; Fig 4). The year effects were observed as positive slopes in the LOESS regressions, which indicate more recent generations were exhibiting larger body lengths than older generations.

Statistical analysis of age cohort 12 was not undertaken because all observations belonged to the same cohort. LOESS regression plotting was also not undertaken for age cohorts 11 and 12 due to insufficient temporal cohorts (n < 3).

## Discussion

It is conceivable that the observed pattern of early phenotypic similarity followed by divergence and late convergence between MR and FR sites reflects the complexity of the main and interactive effects of factors that underlie the impacts that MRs have on the biology and ecology of exploited fish populations on coral reefs. These statistical results must be interpreted with caution given that a posteriori power analysis, used to determine the ability of our statistical analyses to detect significant status effects, identified low statistical power. This indicates reduced ability to determine whether a lack of significant status effect at any given age is, in fact, true or a result of low statistical power, which could stem from small sample sizes, high variance, and/or the given effect size.

Notwithstanding, phenotypic homogeneity during the early life-history stage may reflect larval connectivity, a fitting hypothesis given that *A*. *nigrofuscus* is a broadcast spawner [56] and that coral reef connectivity is predominantly established and maintained through pelagic, larval dispersal [18, 19, 57–59]. This form of connectivity has been shown to establish genetic panmixia between populations and, as a result, suggests that subsequent instances of phenotypic divergence between MRs and FRs are products of post-settlement processes.

Exposure to differing size-selective pressures (e.g. size-selective fishing) between MRs and FRs suggests that individuals inhabiting protected areas are likely to experience heightened survival probabilities while similar sized individuals inhabiting FRs are more likely to experience heightened mortality probabilities. This dichotomy is emphasized for *A*. *nigrofuscus*, whose characteristically fast growth rates place them well within the target range of fishers from an early age [60, 61]. While this hypothesis is supported by the observed significant status effects, which almost exclusively observed the presence of larger body sizes within MRs compared to FRs, high levels of post-settlement connectivity would negate the explanation.

In a meta-analytical investigation of the home ranges and movement patterns of coral reef fishes, Green et al. [62] determined that *A*. *nigrofuscus* exhibit relatively large home ranges that peak at an approximate linear distance of three kilometers. The home ranges of a broad suite of coral-resident fishes, however, have been shown to correlate positively with body size [63], suggesting that the peak three-kilometer home range described by Green et al. [62] might only be exhibited by the largest, and presumably oldest, individuals. As such, it is possible that the phenotypic divergence observed in fish between ages three and six is the result of smaller (younger) individuals’ more restricted home ranges, promoting isolation between MR and FR populations. As individuals continue to grow it becomes more likely that their home range could traverse across the 300-meter boundary space used to separate MR and FR sampling sites, thus enabling both “protected” and “fished” populations to benefit from protection while simultaneously experiencing the threats of FRs. This late onset of post-settlement connectivity could help explain the observed phenotypic convergence at older ages.

In addition to the status effects presented above, significant year effects were observed between ages one and eight, all of which were positive and indicate that younger generations were significantly larger than older generations. If MR populations were exporting individuals with favored life histories, which is highly plausible given the observed spatial results and life history theory of teleosts, then both protected and fished populations could be expected to exhibit gradual enlargements in length-at-age over time as larger size confers greater reproductive output and offspring viability [6, 7]. However, we note that past investigations into the ecophysiology of surgeonfishes have largely found that fishing effort plays little effect, placing greater emphasis on habitat type and quality [60, 64–66]. Definitive answers undoubtedly require metadata on the sampling locations’ physical environment, ecology, and anthropogenic impacts, particularly as they pertain to *A*. *nigrofuscus*.

In conclusion, the longitudinal data procured via the application of the MFBC model provided critical insight into the regional phenotypic responses of *A*. *nigrofuscus* to protection. A broad range of biological and environmental variables have been determined to influence MR performance, illustrated by the variable success of MRs worldwide. As a result, further spatiotemporal analyses across taxa that experience different life histories and across sub-regions that experience different degrees of exploitation are required to determine the ubiquity of these findings. Continued studies confirming the positive conservation and fishery benefits of MRs will remain critical in garnering support for the establishment of MRs globally in the coming decades.

## Supporting information

S1 Fig LOESS regressions of the *A. nigrofuscus* MR and FR age one cohorts between 2003 and 2014. Two-way ANOVA results are shown in the lower left, with significant disparities between year classes (year effects) indicated via year numbers above the regressions.
(TIF)

S2 Fig LOESS regressions of the *A. nigrofuscus* MR and FR age two cohorts between 2004 and 2014. Two-way ANOVA results are shown in the lower left, with significant disparities between year classes (year effects) indicated via year numbers above the regressions.
(TIF)

S3 Fig LOESS regressions of the *A. nigrofuscus* MR and FR age three cohorts between 2005 and 2014. Two-way ANOVA results are shown in the lower left, with significant disparities between year classes (year effects) indicated via year numbers above the regressions.
(TIF)

S4 Fig LOESS regressions of the *A. nigrofuscus* MR and FR age four cohorts between 2006 and 2014. Two-way ANOVA results are shown in the lower left, with significant disparities between year classes (year effects) indicated via year numbers above the regressions.
(TIF)

S5 Fig LOESS regressions of the *A. nigrofuscus* MR and FR age five cohorts between 2007 and 2014. Two-way ANOVA results are shown in the lower left, with significant disparities between year classes (year effects) indicated via year numbers above the regressions.
(TIF)

S6 Fig LOESS regressions of the *A. nigrofuscus* MR and FR age six cohorts between 2008 and 2014. Two-way ANOVA results are shown in the lower left, with significant disparities between year classes (year effects) indicated via year numbers above the regressions.
(TIF)

S7 Fig LOESS regressions of the *A. nigrofuscus* MR and FR age seven cohorts between 2009 and 2014. Two-way ANOVA results are shown in the lower left, with significant disparities between year classes (year effects) indicated via year numbers above the regressions.
(TIF)

S8 Fig LOESS regressions of the *A. nigrofuscus* MR and FR age eight cohorts between 2010 and 2014. Two-way ANOVA results are shown in the lower left, with significant disparities between year classes (year effects) indicated via year numbers above the regressions.
(TIF)

S9 Fig LOESS regressions of the *A. nigrofuscus* MR and FR age nine cohorts between 2011 and 2014. Two-way ANOVA results are shown in the lower left, with significant disparities between year classes (year effects) indicated via year numbers above the regressions.
(TIF)

S10 Fig LOESS regressions of the *A. nigrofuscus* MR and FR age ten cohorts between 2012 and 2014. Two-way ANOVA results are shown in the lower left, with significant disparities between year classes (year effects) indicated via year numbers above the regressions.
(TIF)

S1 Data (CSV)
