# Supplementary figures and images for: A spatiotemporal comparison of length-at-age in the coral reef fish Acanthurus nigrofuscus between marine reserves and fished reefs
Source: PLoS One. 2020 Sep 28;15(9):e0239842. doi: 10.1371/journal.pone.0239842 (PMC7521754; doi:10.1371/journal.pone.0239842)

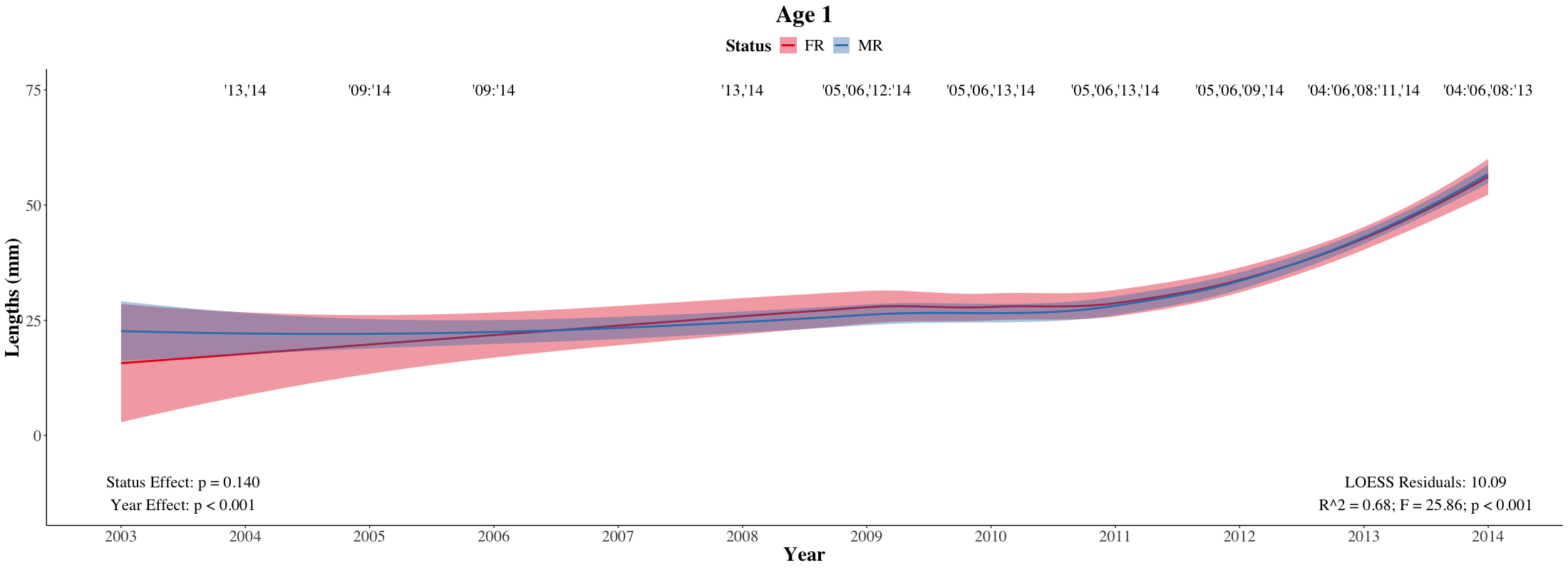

Supplement: S1 Fig — Two-way ANOVA results are shown in the lower left, with significant disparities between year classes (year effects) indicated via year numbers above the regressions. (TIF) [file pone.0239842.s001.tif]

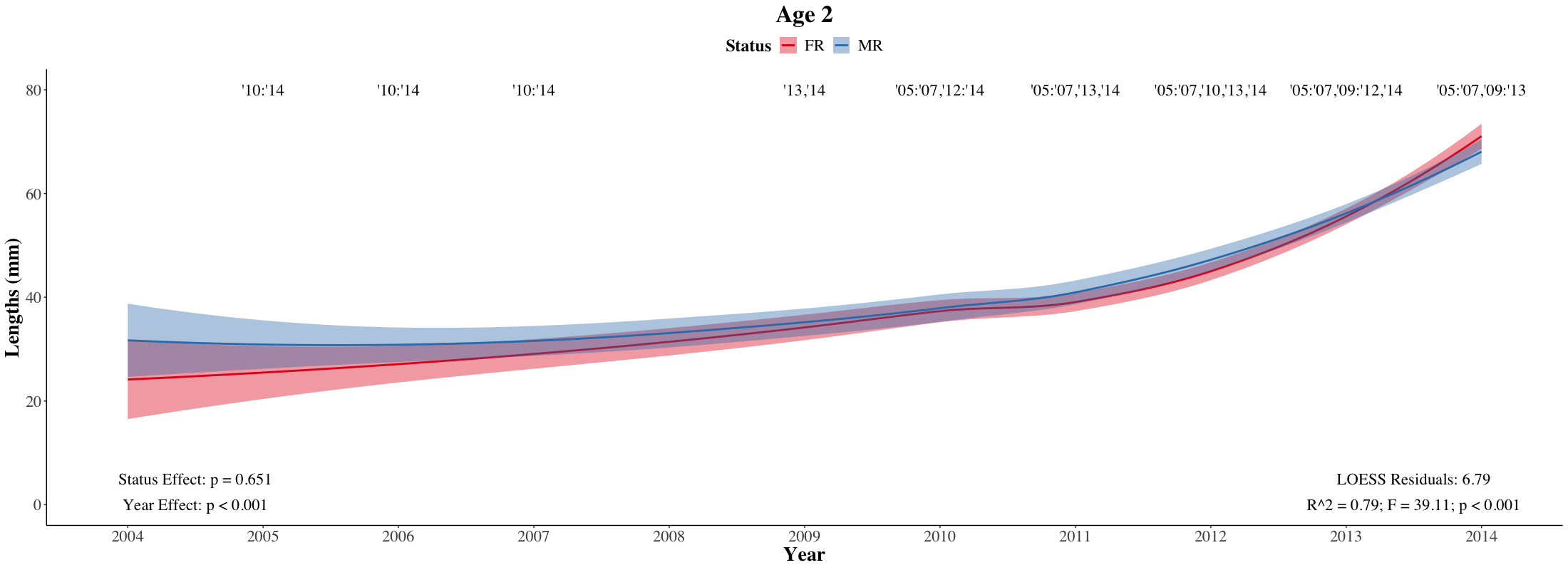

Supplement: S2 Fig — Two-way ANOVA results are shown in the lower left, with significant disparities between year classes (year effects) indicated via year numbers above the regressions. (TIF) [file pone.0239842.s002.tif]

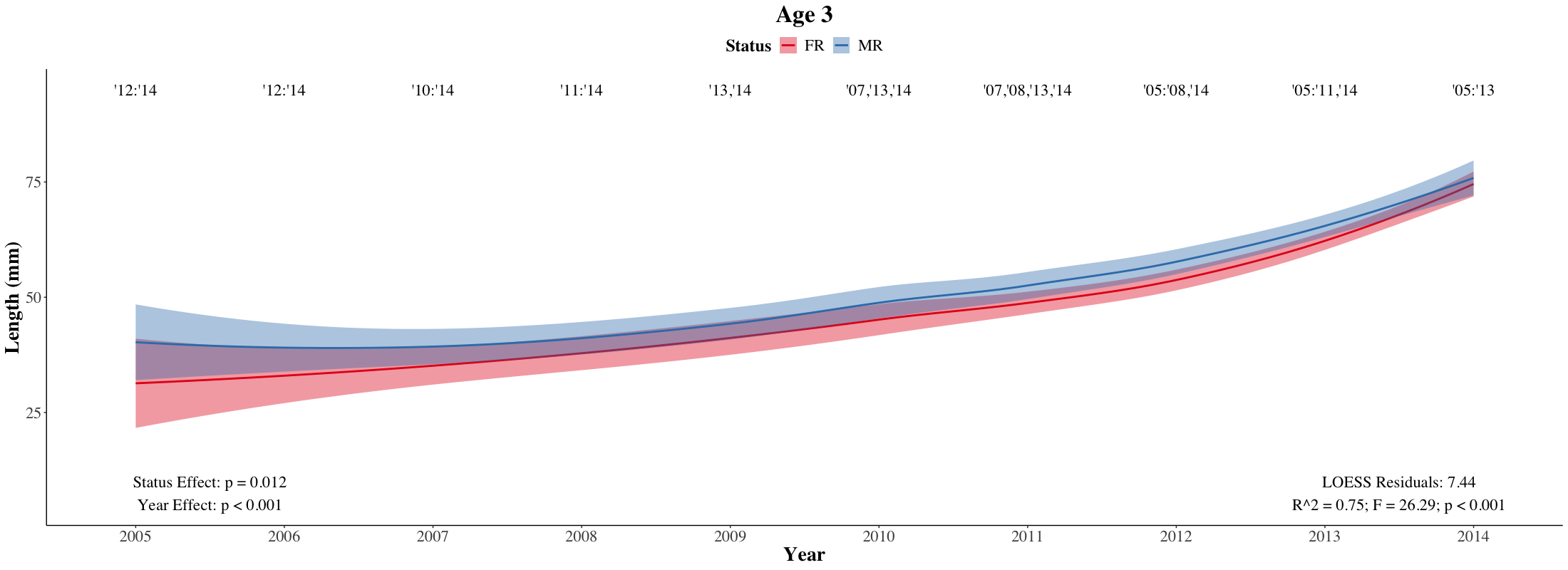

Supplement: S3 Fig — Two-way ANOVA results are shown in the lower left, with significant disparities between year classes (year effects) indicated via year numbers above the regressions. (TIF) [file pone.0239842.s003.tif]

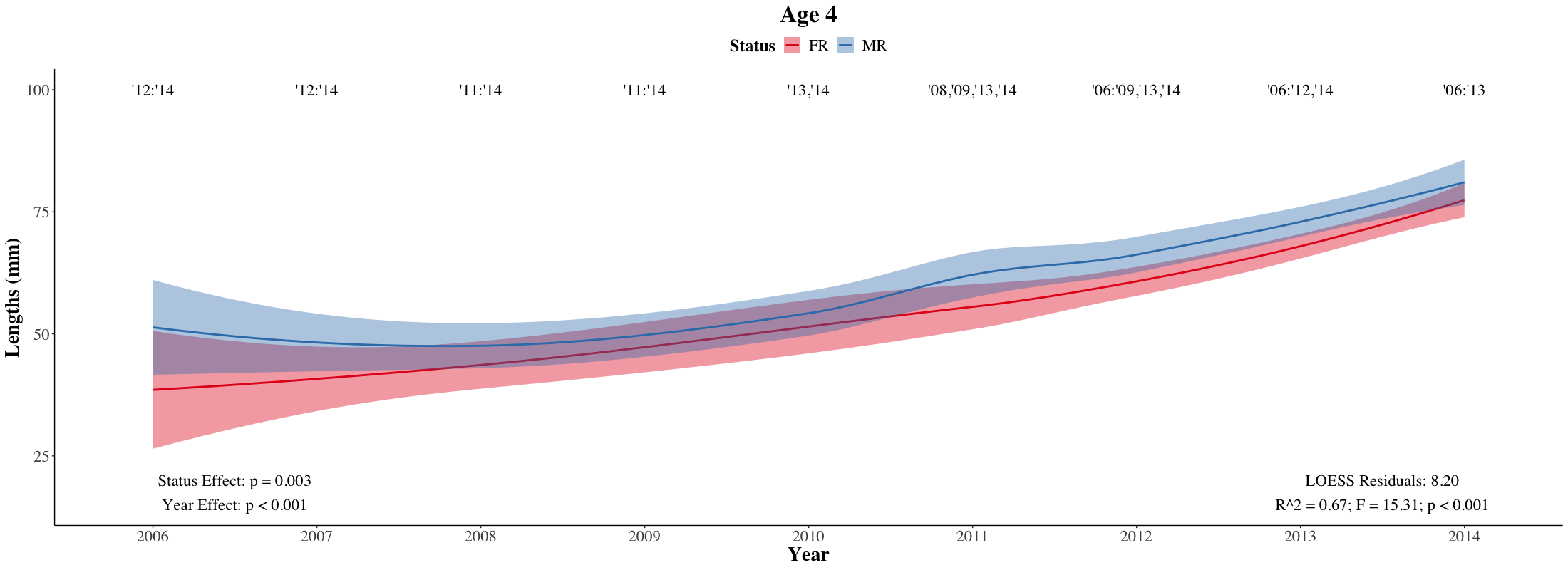

Supplement: S4 Fig — Two-way ANOVA results are shown in the lower left, with significant disparities between year classes (year effects) indicated via year numbers above the regressions. (TIF) [file pone.0239842.s004.tif]

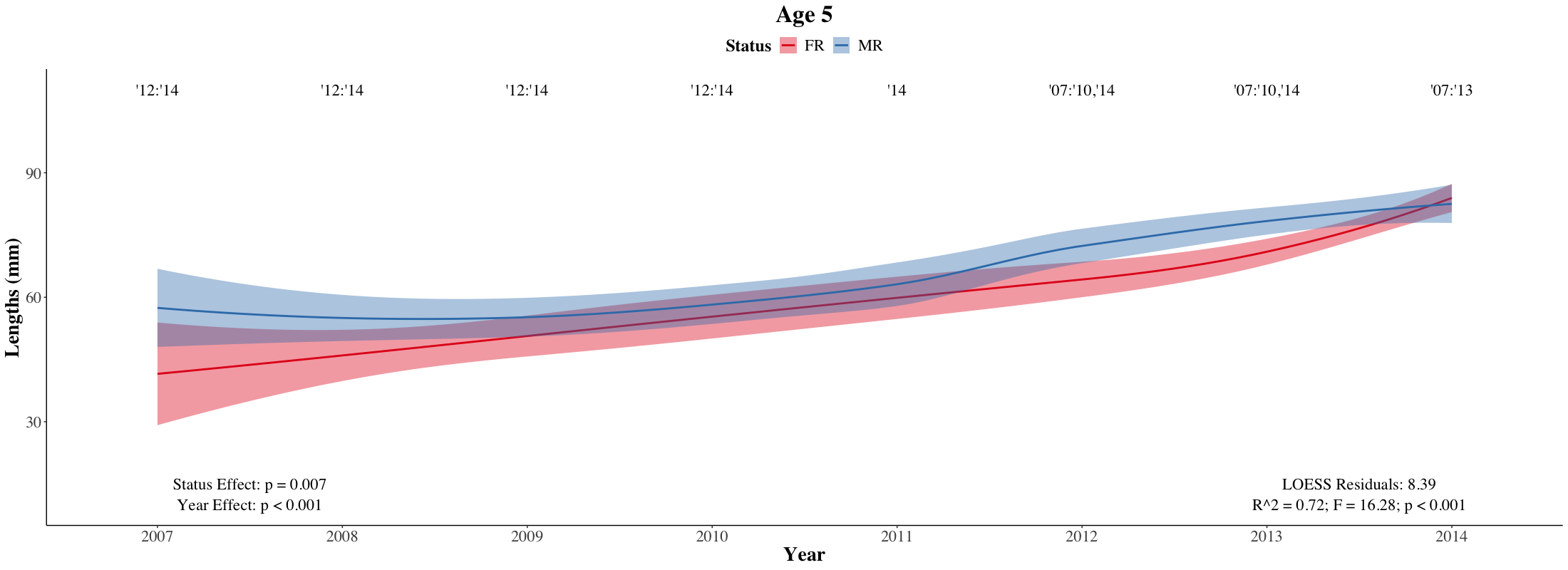

Supplement: S5 Fig — Two-way ANOVA results are shown in the lower left, with significant disparities between year classes (year effects) indicated via year numbers above the regressions. (TIF) [file pone.0239842.s005.tif]

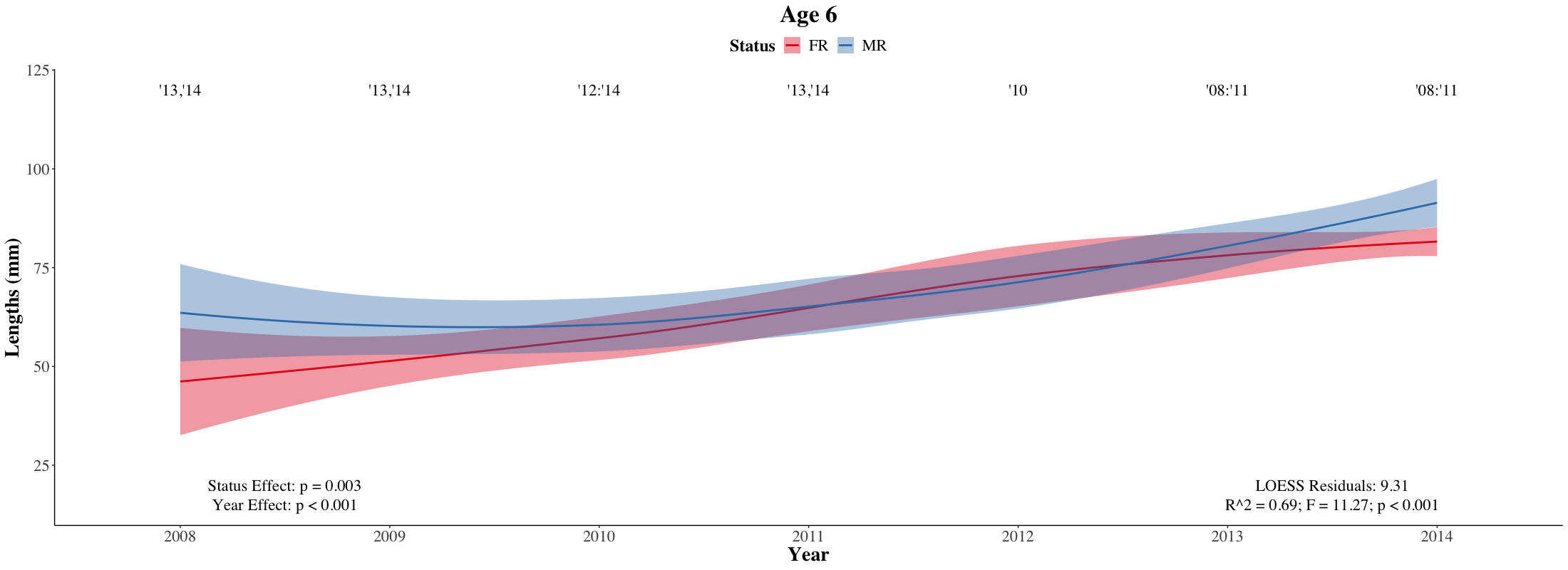

Supplement: S6 Fig — Two-way ANOVA results are shown in the lower left, with significant disparities between year classes (year effects) indicated via year numbers above the regressions. (TIF) [file pone.0239842.s006.tif]

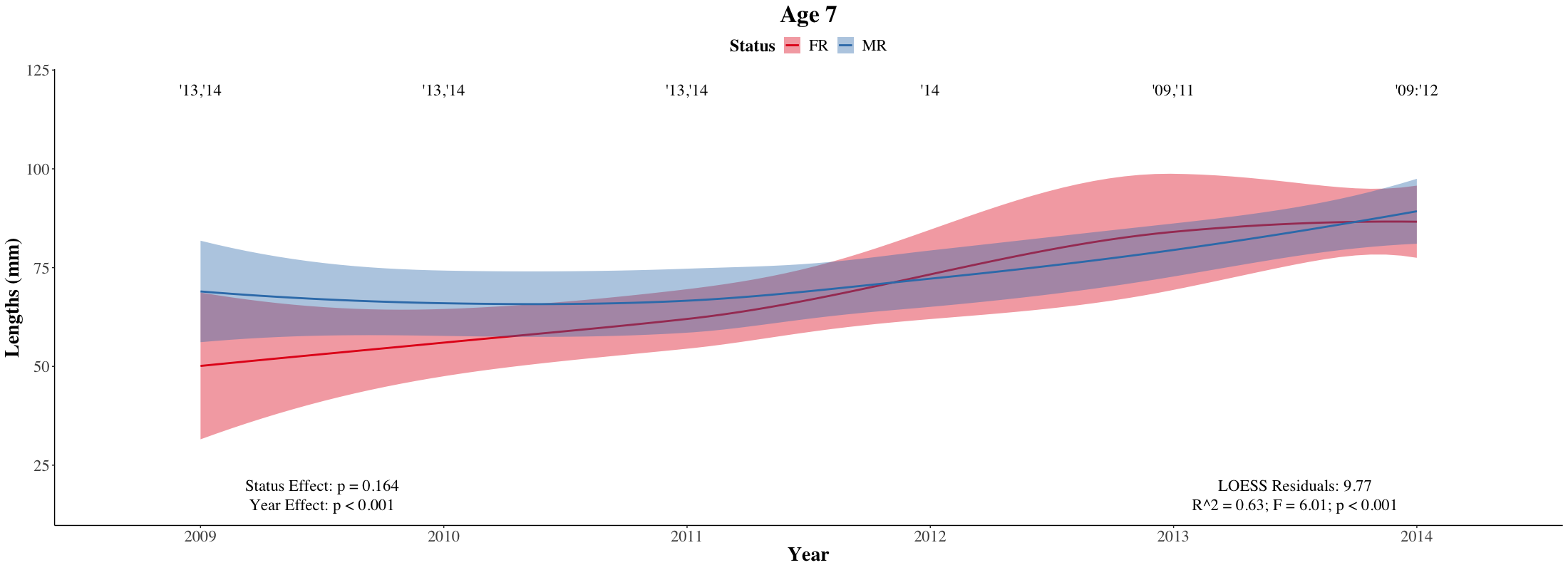

Supplement: S7 Fig — Two-way ANOVA results are shown in the lower left, with significant disparities between year classes (year effects) indicated via year numbers above the regressions. (TIF) [file pone.0239842.s007.tif]

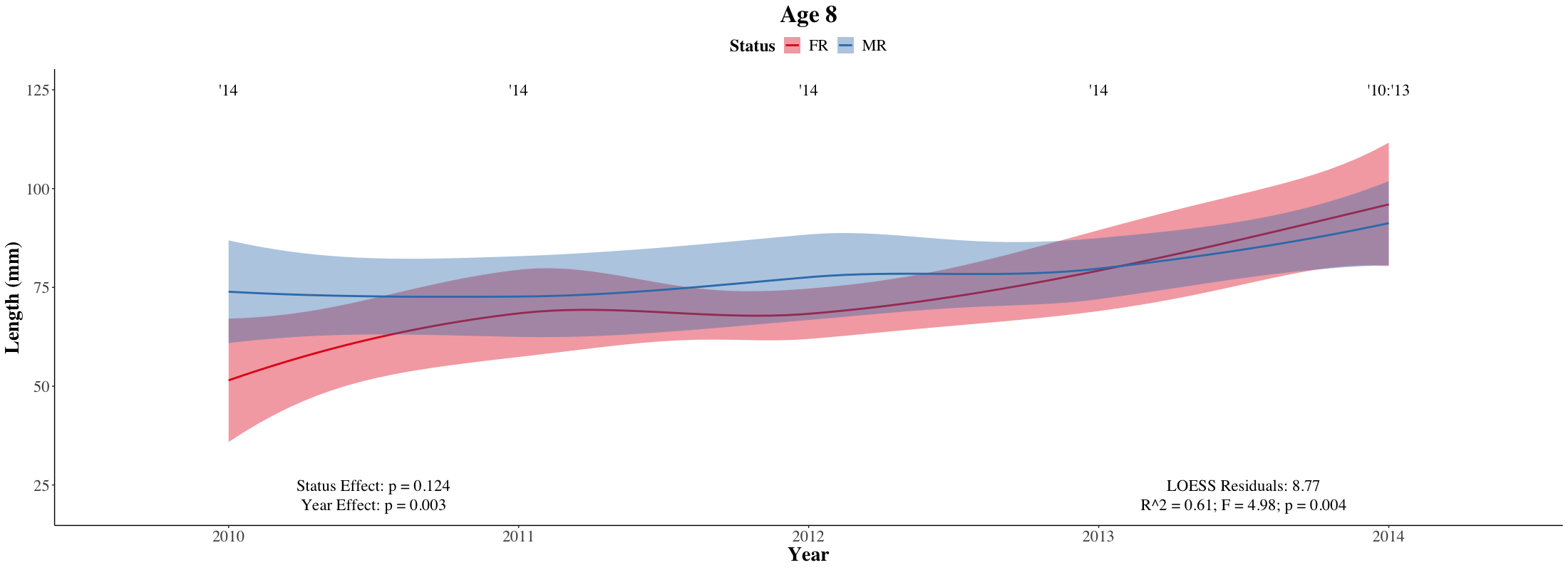

Supplement: S8 Fig — Two-way ANOVA results are shown in the lower left, with significant disparities between year classes (year effects) indicated via year numbers above the regressions. (TIF) [file pone.0239842.s008.tif]

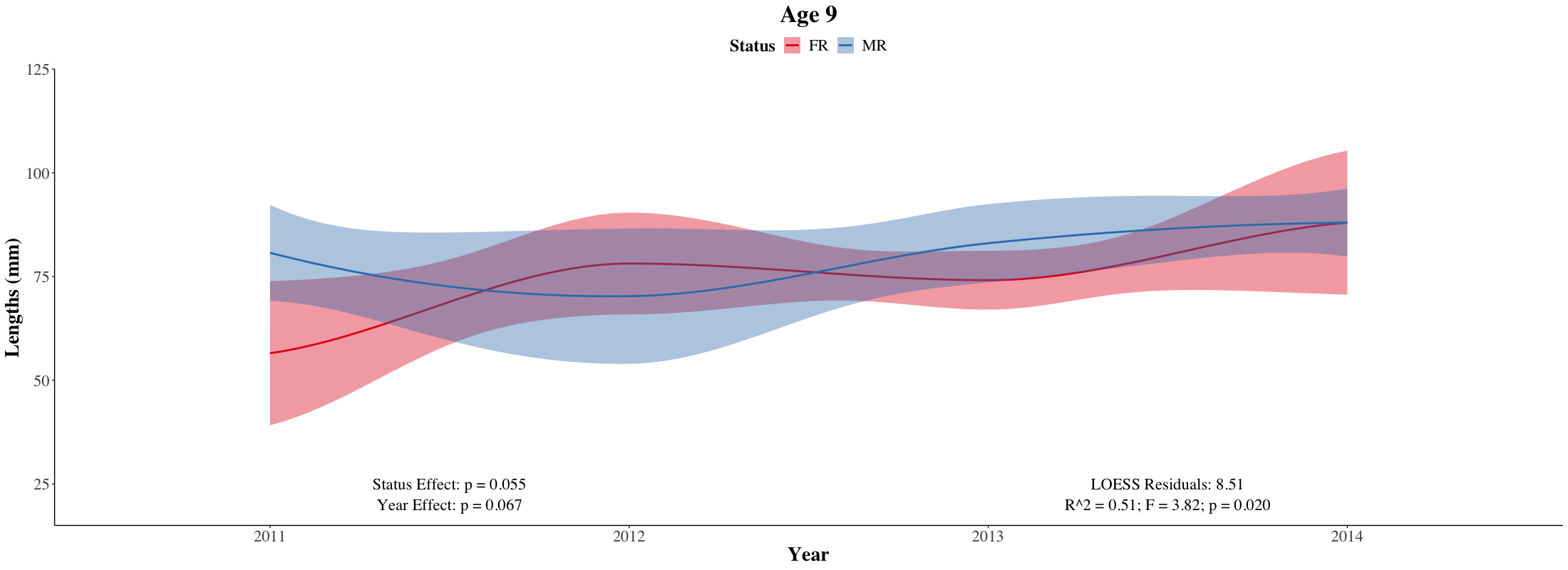

Supplement: S9 Fig — Two-way ANOVA results are shown in the lower left, with significant disparities between year classes (year effects) indicated via year numbers above the regressions. (TIF) [file pone.0239842.s009.tif]

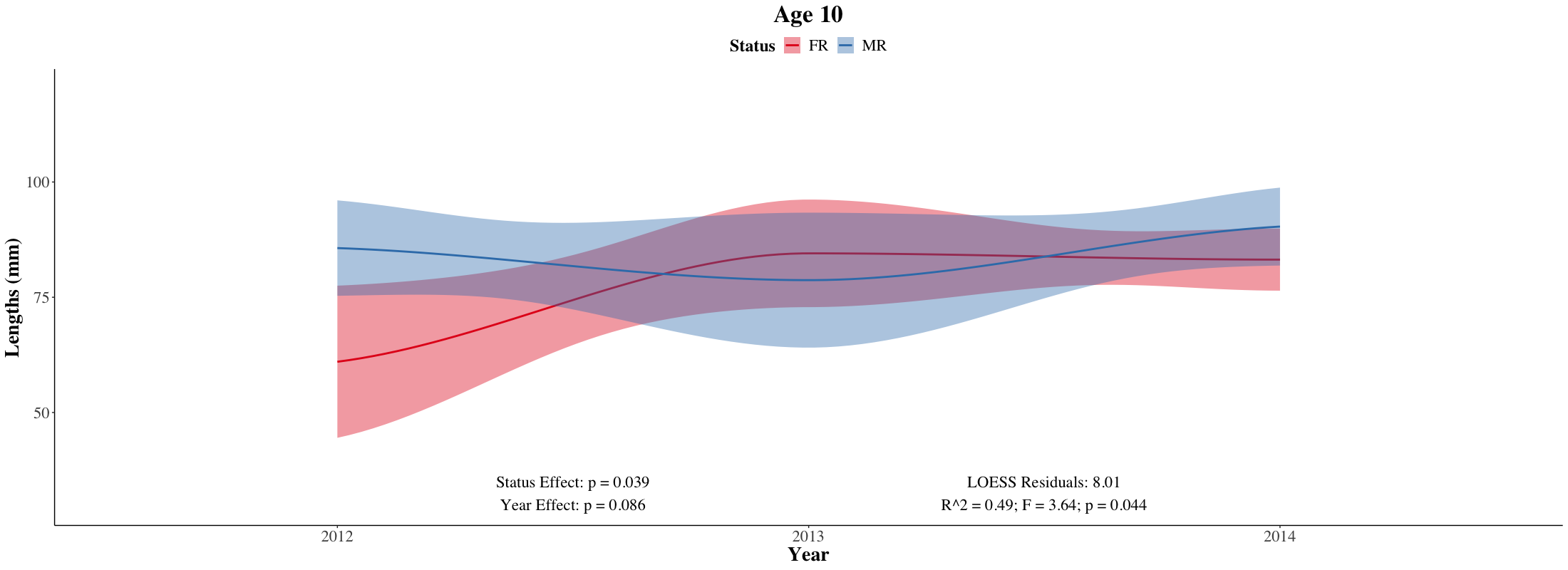

Supplement: S10 Fig — Two-way ANOVA results are shown in the lower left, with significant disparities between year classes (year effects) indicated via year numbers above the regressions. (TIF) [file pone.0239842.s010.tif]
